# Bioinspired Double‐Broadband Switchable Microwave Absorbing Grid Structures with Inflatable Kresling Origami Actuators

**DOI:** 10.1002/advs.202306119

**Published:** 2023-11-30

**Authors:** Zhong Zhang, Hongshuai Lei, Shengyu Duan, Zeang Zhao, Mingji Chen, Changxian Wang, Daining Fang

**Affiliations:** ^1^ Beijing Key Laboratory of Lightweight Multi‐functional Composite Materials and Structures Institute of Advanced Structure Technology Beijing Institute of Technology Beijing 100081 P. R. China; ^2^ School of Materials Science and Engineering Nanyang Technological University Singapore 639798 Singapore; ^3^ State Key Laboratory for Turbulence and Complex Systems College of Engineering Peking University Beijing 100871 P. R. China

**Keywords:** bioinspired structure, bistable state, broadband microwave absorption, Kresling origami, tunable performance

## Abstract

Tunable radar stealth structures are critical components for future military equipment because of their potential to further enhance the design space and performance. Some previous investigations have utilized simple origami structures as the basic adjusting components but failed to achieve the desired broadband microwave absorbing characteristic. Herein, a novel double‐broadband switchable microwave absorbing grid structure has been developed with the actuators of inflatable Kresling origami structures. Geometric constraints are derived to endow a bistable feature with this origami configuration, and the stable states are switched by adjusting the internal pressure. An ultra‐broadband microwave absorbing structure is proposed with a couple of complementary microwave stealth bands, and optimized by a particle swarm optimization algorithm. The superior electromagnetic performance results from the mode switch activating different absorbing components at corresponding frequencies. A digital adjusting strategy is applied, which effectively achieves a continuously adjusting effect. Further investigations show that the proposed structure possesses superior robustness. In addition, minimal interactions are found between adjacent grid units, and the electromagnetic performance is mainly related to the duty ratio of the units in different states. They have enhanced the microwave absorbing performance of grid structures through a tunable design, a provided a feasible paradigm for other tunable absorbers.

## Graphical abstract

A novel bioinspired double‐broadband switchable microwave absorbing structure has been developed with actuators of bistable inflatable Kresling origami structure. The optimized absorber possesses complementary low‐frequency and high‐frequency microwave absorbing bands with different actuator states and achieves a robust variation of electromagnetic performance with a digital adjusting strategy. The investigation provides a feasible paradigm for greater microwave stealth capacity with structural reconfiguration.

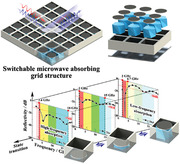

## 1 Introduction

As the most basic object in the universe, the electromagnetic wave is more and more widely used in the information age, and the control technique has become a significant scientific subject in recent years.^[^ ^1^ ^]^ To satisfy the ever‐increasing demands, electromagnetic materials are being vigorously developed,^[^ ^2^ ^]^ such as negative refractive index,^[^ ^3^ ^]^ electromagnetic cloak,^[^ ^4^ ^]^ and high electromagnetic loss.^[^ ^5^ ^]^ Herein, the perfect absorber is a type of metamaterial or metastructure that possesses the capacity to greatly reduce the reflected electromagnetic waves in a specific frequency range.^[^ ^6^ ^]^ The corresponding research has been pursued for decades but few expand the absorbing band to a satisfying degree.^[^ ^7^ ^]^ During the research, various microwave absorbers were developed, which can be classified according to the spatial configuration as 2D^[^ ^8^ ^]^ and 3D absorbers.^[^ ^9^ ^]^ In general, 2D absorbers possess commendable absorbing performance at a specific frequency due to electromagnetic resonance, while 3D absorbers possess superior absorbing performance in high‐frequency ranges due to the continuous electromagnetic loss in the transmission process.^[^ ^7a^ ^]^ The electromagnetic performance is fixed once manufactured, especially the absorbing band with a reflectivity of less than −10 dB. However, the conventional design concept to increase the fixed bandwidth limits the development of these absorbers. For practical electromagnetic radars, the operating frequency is positively correlated to the detecting precision and negatively to the maximum detecting range.^[^ ^10^ ^]^ The low‐frequency electromagnetic wave is utilized for long‐range surveillance and the high‐frequency is for short‐range tracking. Therefore, it is feasible to expand the electromagnetic stealth performance by tuning the absorbing band according to the distance between the object and radar stations. The tunability becomes an attractive subject for metamaterial absorber development.^[^ ^11^ ^]^

As for the tuning approaches, recent investigations focus on four dominating aspects,^[^ ^11^, ^12^ ^]^ including lumped elements,^[^ ^13^ ^]^ phase‐change materials,^[^ ^14^ ^]^ graphene materials,^[^ ^15^ ^]^ and mechanically reconfigurable structures.^[^ ^16^ ^]^ The lumped elements are particularly suitable for metasurfaces, which are generally composed of various metal patterns connected with diodes or other controllable lumped electronic elements. As electromagnetic waves propagate to these metasurfaces, the resonance occurs between metallic patterns, resulting in a significant electric current and thermal loss on the lumped elements. By using a tunable semiconductor as the lumped elements, the resonance and loss effect can be conveniently changed by a bias voltage. However, it is quite hard for these metasurfaces to achieve broadband microwave absorption.^[^ ^17^ ^]^ Phase‐change materials, such as liquid crystals and VO_2_, generally possess a certain electromagnetic wave absorbing capacity, which can be utilized in both 2D and 3D absorbers. However, the phase‐changing mechanism is largely related to the ambient temperature, resulting in obstacles for practical applications.^[^ ^14c^ ^]^ Graphene, with a few atomic layers, is a promising semiconducting material. Its electromagnetic performance can be adjusted by a bias voltage and is more suitable for the THz frequency range due to the thickness restriction.^[^ ^15a^ ^]^ For mechanically reconfigurable structures, the traditional tuning method is to apply external forces and change the thickness of specimens utilizing the elasticity of materials.^[^ ^16c^ ^]^ Recently, origami configuration has been developed as the main tuning component and has become increasingly attractive with the benefit of large variations between 2D and 3D configurations.^[^ ^18^ ^]^ Some simple origami absorbers have been developed from the Miura origami structure with frequency selective surface (FSS)^[^ ^19^ ^]^ and polygon grid structure.^[^ ^20^ ^]^ These origami absorbers exhibit remarkable tuning effects on electromagnetic performance.^[^ ^21^ ^]^ However, the deformation process is actuated by changing the in‐plane coverage area. Correspondingly, the background panel has to change with configuration, which is not suitable for some specific applications, such as the outside surface of military equipment. In addition, these investigations are more concerned with the intensity of absorbing peaks, while the bandwidth is far from satisfying the demand for radar stealth.^[^ ^22^ ^]^ Therefore, a practical broadband tunable absorber is urgently needed.

**Figure 1:**
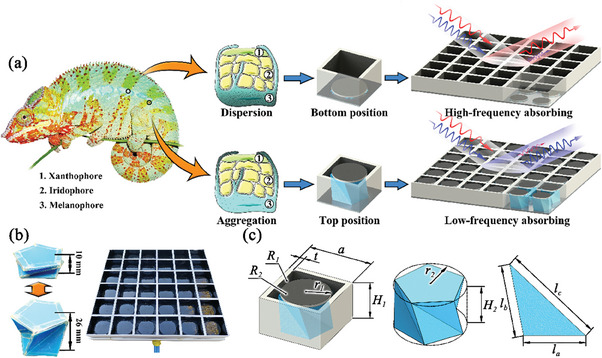
Schematic illustration of the broadband switchable microwave absorber: a) Biomimetic mechanism and expected microwave absorbing effect, b) Fabricated Kresling origami component and assembled broadband switchable microwave device, and c) Structural parameters in the design process.

To this end, we propose a novel broadband switchable microwave absorber inspired by the color changes in chameleons.^[^ ^23^ ^]^ The color‐changing function in chameleon skin is a synergistic process involving three different kinds of chromatophores, as xanthophores, iridophores, and melanophores. The process can be classified as the gradual intraspecific color variation and rapid brightness color variation according to the response speed and color gamut. The gradual intraspecific color variation results from the change of closely packed guanine crystals in xanthophores and iridophores. The rapid brightness color variation results from the dispersion/aggregation of melanosomes in melanophores. Correspondingly, the microwave absorbing device is designed as a combination of impedance‐type square grid structure, that is a kind of typical 3D absorber, and circular resistance piece arrays, which acted as FSS, that is a kind of 2D metasurface (**Figure** **1**a). The square grid structure corresponds to the closely packed guanine crystals, which provide a basic microwave‐absorbing performance for the whole device. The circular resistance pieces correspond to the melanosomes, which change the absorbing performance by fluctuating up and down in the grid units. To control the position of circular resistance pieces, the Kresling origami structure is utilized as an actuator, which can easily change its height by air inflation. Simultaneously, it can maintain the states without a continuous external force by introducing the bistability feature.^[^ ^24^ ^]^

In the preliminary design concept, the main active device is designed to switch between a resistance piece array and an impedance‐type grid structure by changing the position of these pieces between the top and bottom. Herein, the impedance‐type grid structure possesses superior absorption performance in the high‐frequency range but worse in the low‐frequency range.^[^ ^25^ ^]^ On the other hand, the resistance piece array can be flexibly designed in a specific frequency range to recover the shortage of impedance‐type grid structure and achieve broadband electromagnetic absorption. As the verification, a series of specimens is optimized, fabricated, and tested. When the resistance pieces are at the top of the grid structure, the absorber realizes a superior microwave absorbing band in a low‐frequency range (2–4.7 GHz) and a certain reflecting band in a high‐frequency range (4.7–18 GHz). When the resistance pieces are at the bottom, the absorber realizes an ultra‐wide absorbing band in the high‐frequency range (3.4–18 GHz) and a certain reflecting band in the low‐frequency range (2–3.4 GHz). The complementary absorbing performance identifies with the ideal design objective, indicating that the proposed broadband switchable absorbing device takes full advantage of 2D/3D absorbing units and broadens the absorbing band with practical significance. This study paves a new way for similar tunable microwave absorbing devices.

## 2 Results and Discussion

In the design process, the proposed broadband switchable microwave absorbing device is composed of three primary components: an impedance‐type square grid structure, a series of circular resistance pieces, and corresponding bistable Kresling origami airbags. In addition, a perfect electrical conductor (PEC) is set as the background. The impedance‐type square grid structure is a kind of typical high‐porosity microwave absorber with superior load‐bearing capacity and high‐frequency microwave absorption.^[^ ^9^, ^25^ ^]^ Therefore, it serves as the basic framework for the whole device. The periodically arrayed circular resistance pieces act as a kind of FSS, the position of which can significantly change the microwave absorbing performance of the grid structure.^[^ ^26^ ^]^ Therefore, the resistance pieces are set as the modulator. The Kresling origami structure possesses bistable features and a stable deformation process. Therefore, a series of Kresling origami airbags is introduced as the actuator to control the position of resistance pieces. The airbags are hermetic and can be inflated by an air pump. When inflated, the origami airbag expands and keeps an approximate polygon prism configuration. When exhausted, the origami airbag folds to a 2D surface. In this design, the stable states of a Kresling origami are set corresponding to the top and bottom positions of the resistance pieces, expecting to maximize the variation of microwave absorbing performance.

### 2.1 Bistable Feature of Kresling Origami

As Kresling origami is an important component, its geometric configuration is first investigated. The structure parameters are exhibited (Figure 1c) and the origami height (*H*_2_) is the critical parameter related to electromagnetic performance, which is determined by the triangle side panels and polygonal terminal panels.^[^ ^18b^ ^]^ Therefore, an investigation of the Kresling origami geometrical configuration is carried out (Note S1, Supporting Information). According to the geometrical relationship of the configuration in folded and expanded states, the side length values of triangle panels should satisfy the geometric constraint of these configurations, denoted as:

(1) $$\frac{l_{b}^{2}+l_{c}^{2}-l_{a}^{2}}{2l_{b}l_{c}}=\frac{l_{b}^{2}+l_{c}^{2}-l_{a}^{2}-2H_{2}^{2}}{2\sqrt{\left(l_{b}^{2}-H_{2}^{2}\right)\left(l_{c}^{2}-H_{2}^{2}\right)}}=cos\frac{\pi }{n}$$

where *l_a_*, *l_b_* and *l_c_* are the side length values of the triangle panel, and *n* denotes the number of polygonal panel sides. Simultaneously, the side length of terminal panels (*l_a_*) can be expressed as:

(2) $$l_{a}=2r_{2}\times sin\frac{\pi }{n}$$

where *r*_2_ refers to the circumradius of polygonal panels. Setting the origami height (*H*_2_), side number (*n*), and polygonal circumradius (*r*_2_) as known quantities, the side length values of triangle side panels (*l_a_*, *l_b_*, *l_c_*) are obtained by substituting Equation (2) into Equation (1). A tension‐twist coupling behavior is observed when the upper polygon panel moves from the bottom position to the top due to the chirality feature (Note S1, Supporting Information). This behavior is temporarily eliminated in this study by using circular resistance pieces, but it can be utilized in a spatial phase converter for further studies. The top polygon panel turns a twist angle (θ) can be given as:

(3) $$\theta =2\left(arccos\frac{l_{a}^{2}+l_{c}^{2}-l_{b}^{2}}{2l_{a}l_{c}}-arccos\frac{l_{a}^{2}+l_{c}^{2}-l_{b}^{2}}{2l_{a}\sqrt{l_{c}^{2}-h_{2}^{2}}}\right)$$

**Figure 2:**
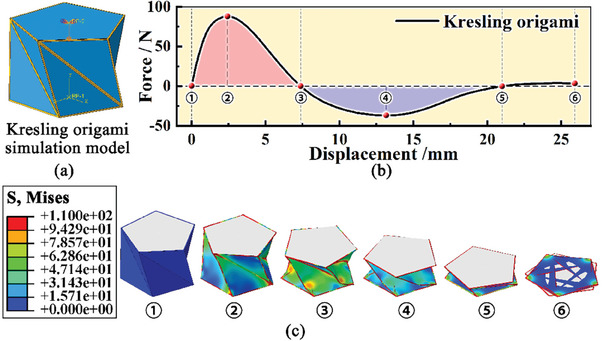
Verification of the bistable feature for Kresling origami structure: a) Simulation model with shell elements, b) Axial compression performance, and c) The Mises equivalent stress distribution diagrams during compression.

As for the stable feature, the principle of minimum potential energy is utilized for evaluation. The polygonal terminal panel is assumed to be rigid, and the height variation totally results from the deformation of triangle side panels. According to the geometric constraint in Equation (1), when the height of Kresling origami equals to 0 or *H*_2_, the side length values of the triangle panels are equal to *l_a_*, *l_b_* and *l_c_*, which means these side panels keep flat, the strain energy is zero and the origami structure remains stable. Otherwise, when the height varies, the side length values are no longer equal to the original, which means the side panels start to inflect, the strain energy increases and the structure becomes unstable. Therefore, the Kresling origami possesses bistable features. To verify this feature, a compression characteristic is qualitatively analyzed with finite element models (FEM) (Note S2, Supporting Information). A series of structural parameters is utilized according to the geometric constraints (Table S1, Supporting Information). In the whole compression process, the height of the Kresling origami model keeps decreasing from 26 to 0 mm. The corresponding compressive force is recorded and plotted (**Figure** **2**).

The compressive curve is divided into three distinct segments. In the first segment, the reaction force remains positive and does positive work. The force ascents rapidly reaches a peak value of 57.0 N at ≈2.41 mm, and descends to 0 N at ≈18.9 mm. In the second segment, the reaction force remains negative and does negative work. The compressive force descends to −17.0 N at ≈12.5 mm and increases to 0 N at ≈5.1 mm. In the third segment, the compressive force slightly increases from 0 N to a small positive value of 3.4 N. The Mises equivalent stress distribution diagrams of the origami model at six specific points during the compression are also exhibited (Figure 2), corresponding to the work done by external forces.

It should be noted that the second stable state is designed at a compressive displacement of 26 mm. However, the stable state is achieved prematurely in simulation, although the contact between side panels is neglected. This distinction may result from the deformation of boundary strips between these side panels. According to the stress distribution diagrams, the majority of side panels are nearly strain‐free at 26 mm, while the boundary strips are largely deformed, which slightly generates additional strain energy and influences the stable point.

### 2.2 Pneumatic Experiments of Kresling Origami

**Figure 3:**
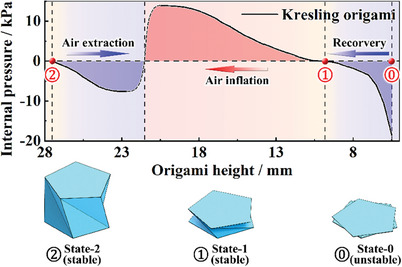
Mechanical response of Kresling origami for flat compression and air inflation.

In order to observe the deformation process, a series of Kresling origami airbags is fabricated with PVC sheets and PE films manually. Pneumatic experiments are conducted with these origami airbags. According to the experimental results, the height of the Kresling origami specimen slightly deviates from the design. When origami is stably expanded (marked as State‐2), the height is 28 mm (26 mm in design). When the origami is stably folded (marked as State‐1), the height descends to 10 mm (0 mm in design). If negative pressure is applied (marked as State‐0), the height decreases up to the minimum of 4 mm (0 mm in design). The distinction between practical specimen and design mainly results from the neglect of the panel thickness and mechanical interference. Taking these states as the initial points, three independent pneumatic experiments are conducted, including air extraction tests (Movie S1, Supporting Information), air inflation tests (Movie S2, Supporting Information), and negative pressure recovery tests (Movie S3, Supporting Information). The obtained three height–pressure curves are integrated to obtain a relationship between internal pressure and structure height (**Figure** **3**). It should be noted that during air extraction and inflation experiments, the origami specimen exhibits sudden deformations at the height of ≈22.5 mm (−7.6 kPa, relative pressure) and 20.5 mm (14 kPa, relative pressure) respectively, which implies the buckling at the side panels of these specimens. The ability to resist deformation starts to decrease at these points, resulting in a positive feedback process and accelerating the deformation process. The actual internal pressure should be continuous and between −7.6 and 14 kPa. Therefore, an estimated curve is added between the breaking points with the dotted line, referring to the deformation velocity in tests.

According to the integrated height–pressure curves, the critical switching pressure in the air extraction process is slightly less than the inflation process. This result is not completely consistent with the simulation. This probably results from the difference in the force‐applying methods. In simulations, the external forces are barely applied on the top and bottom panels, which remain the same during the whole process. In pneumatic experiments, the internal pressure is utilized to drive the specimen. The air pressure is applied not only on the top and bottom panels but also on the side panels. The applying area in State‐2 is much larger than in State‐1. Therefore, the critical pressure in the first segment is lower than the second segment. Besides, negative pressure recovery tests are conducted instead of air inflation tests to capture pneumatic performance in the third segment. An excessive negative internal pressure is first applied to make the specimen completely folded (State‐0). Then, the pressure is gradually recovered and the corresponding structure height is recorded. It can be observed that the test data in the third segment are perfectly connected with the second segment. The structure height decreases with the internal pressure and approaches the limitation of 4 mm. In summary, the air inflation tests verify the bistability of the fabricated Kresling origami airbag. The specimens possess a pair of stable states at the structure height of 28 and 10 mm. Although the height in folded stable state deviates from the design, the structure height can further decrease to 4 mm by applying an extra negative internal pressure, which satisfies the design requirements. An investigation of the durability of the inflatable Kresling origami actuator is also conducted as a reference (Note S3, Supporting Information). For future practical applications, industrialized fabrication technology and more suitable materials can be utilized to improve the performance to conform the design results more precisely.

### 2.3 Parameter Design and Optimization

In order to adequately utilize the bistable feature of Kresling origami airbags, the particle swarm optimization algorithm is established to obtain a series of superior structural parameters (Note S4, Supporting Information). The optimization parameters include unit period (*a*), wall thickness (*t*), grid sheet resistance (*R*_1_), circular sheet resistance (*R*_2_), circular sheet radius (*r*_1_) and origami height (*H*_2_) (Figure 1c). A series of numerical simulation models in both expanded and folded states are established in CST Studio Suite to obtain the reflectivity curves (Note S5, Supporting Information). Then, the numerical calculation results are extracted through an interface program and used in optimization. The main objective is to develop a switchable microwave absorber that possesses opposite microwave absorbing performances in different states and achieves the maximal combined absorbing band, ranging from 2 to 18 GHz. Herein, a customized fitness function with a piecewise linear form is utilized for these purposes. The optimization problem can be formulated as follow:

(4) $$\left\{\begin{array}{cc} Find & a,t,R_{1},R_{2},r_{1},H_{2}\\ s.t. & \begin{array}{c} \begin{array}{c} 20\leq a\leq 50\\ 0.5\leq t\leq 2.5\\ 200\leq R_{1}\leq 1000 \end{array}\\ \begin{array}{c} 10\leq R_{2}\leq 300\\ 0.7\left(a-t\right)\leq r_{1}\leq 0.98\left(a-t\right)\\ 5\leq H_{2}\leq 15 \end{array} \end{array}\\ Maximize\prod_{i\;=\;1}^{2} & \int_{f_{b}}^{2}F_{li}\left(f_{i}\left(x\right)\right)dx\cdot \int_{18}^{f_{b}}F_{hi}\left(f_{i}\left(x\right)\right)dx \end{array}\right.$$

where *f_b_* refers to the frequency of breakpoints, *F_li_* and *F_hi_* represent fitness functions, *f_i_* denotes the reflectivity of the whole absorber and the subscript *i* represents the state number (1 refers to the folded state and 2 refers to the expanded state). After global optimization, a series of structural parameters are obtained (Table S2, Supporting Information).

### 2.4 Switchable Microwave Absorbing Effect

To verify the availability of the proposed broadband switchable microwave absorber, a series of circular resistance pieces and impedance‐type square grid structures are fabricated with conductive graphite ink and polyamide using screen printing and selective laser sintering method. An integrated structure is assembled by prefabricated origami airbags, circular resistance pieces, and an impedance‐type square grid structure (Figure 1b). As full‐size Kresling origami airbags are rather complex and difficult to fabricate in the laboratory, the whole integrated structures are scaled up with a factor of 2, according to the scaling effect^[^ ^27^ ^]^ (Figure S7, Supporting Information). For the convenience of comparison, the subsequent experimental data are modified by blue‐shifting the reflectivity curves with a factor of 2 to match the design and simulation results. To further improve the performance of the origami airbags, some professional manufacturing techniques can be utilized, such as the injection molding process. The low‐stiffness creases can be integrally fabricated with the high‐stiffness panels and distinguished by setting different thicknesses, which will effectively scale down the airbag size to satisfy the design results. According to the pneumatic experiments of the Kresling origami actuator, the microwave absorbing performance of broadband switchable microwave absorbers is measured in three states, including completely folded state (State‐0), stably folded state (State‐1), and stably expanded state (State‐2). In the microwave experiments, the incident electromagnetic energy is split into three segments, that is absorption, reflection, and transmission. Herein, the transmissivity is proved negligible in simulation (Figure S8, Supporting Information). The microwave absorbing effect is directly evaluated by the reflectivity. In general, if the reflectivity of an absorber is less than −10 dB, the absorber is deemed to possess an electromagnetic stealth feature in the given frequency range.^[^ ^28^ ^]^

**Figure 4:**
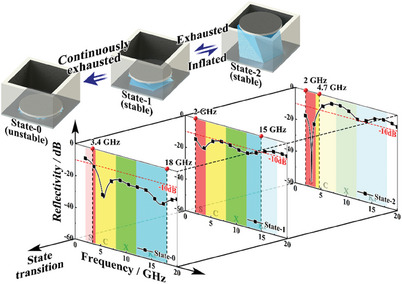
Variation of microwave absorbing performance during the state switching process.

**Figure** **4** shows the variation of structural configurations and measured microwave reflectivity curves for different states in the frequency range of 2–18 GHz. As illustrated in Section 2.2, these states can be conveniently switched by inflating or exhausting (Movie S5, Supporting Information). In State‐0, the specimen exhibits a broadband microwave absorbing feature in the frequency range of 3.4–18 GHz. The reflectivity curve of a bare impedance‐type grid absorber is also measured and compared with the integrated absorber in State‐0 (Figure S9, Supporting Information). The results reveal that these reflectivity curves are nearly coincident and only slightly separate with increasing frequency. It indicates that the outstanding high‐frequency performance benefits from the component of the impedance‐type grid structure. The equivalent electromagnetic parameters of these gird structures are comparable to the Debye model and can be flexibly adjusted by changing structural parameters.^[^ ^25^ ^]^ The distinct in high‐frequency range results from circular resistance pieces and the penetrability of microwaves with different wavelengths.

In State‐1, the reflectivity curve exhibits obvious variation compared with State‐0. The first absorbing peak (reflectivity valley) red‐shifts from 5.6 to 3.4 GHz and enhances the microwave absorbing performance in the low‐frequency range. Meanwhile, the microwave absorbing absorption effect in the high‐frequency range integrally decreases and the microwave absorbing band prematurely terminates at 15 GHz. In State‐2, the variation tendency exhibits the same as in State‐1. Furthermore, the first absorption peak shifts to 3.2 GHz, and the microwave absorbing effect in the high‐frequency range decreases, presenting a narrow but strong (−56.3 dB) microwave absorbing band in the frequency range of 2–4.7 GHz. This remarkable variation results from the electromagnetic adjusting capacity of circular resistance pieces. These pieces are arrayed periodically and act as FSS in the grid structure. The FSS can be utilized as a kind of spatial filter, which can selectively absorb, reflect, and transmit electromagnetic waves according to the frequency. When these pieces are located at the top of grid units, the incident electromagnetic waves are first filtered by the FSS to determine whether they will propagate to the subsequent grid structure or not. To investigate the specific effect of these resistance pieces, the electromagnetic performance of the resistance array, including reflectivity, transmissivity, and absorptivity, is calculated using simulation analysis (Figure S10, Supporting Information). The result shows that the absorptivity maintains a nearly constant value in the whole frequency range. The reflectivity is rather small in the low‐frequency range and maintains at a high level in the high‐frequency range, which is contrary to the transmissivity. Hence, the resistance array acts as a wave‐transmitting material in the low‐frequency range, which enhances the microwave absorbing performance, while acting as a wave‐shielding material, which restrains the microwave from transmitting to the grid structure and weakens the microwave absorbing performance. Therefore, the integrated absorber in State‐2 only achieves a narrow but strong microwave absorption in the low‐frequency range. In general, the specimen exhibits a remarkable switching effect on the microwave absorbing performance between State‐0 and State‐2. The absorbing and reflecting bands are directly opposite to each other, which is consistent with the design objective and verifies the validity of the optimization program. It should be noted that only the frequency range of 2–18 GHz is considered in this study. The specimen is equipped with a much broader absorbing band in the high‐frequency range in State‐0 and low‐frequency range in State‐2. In addition, another optimization design is conducted setting stable states (State‐1 and State‐2) as the desired states (Note S6, Supporting Information). The additional optimization results show a similar switching effect, but the reflectivity in the high‐frequency range slightly exceeds the expected value of −10 dB.

**Figure 5:**
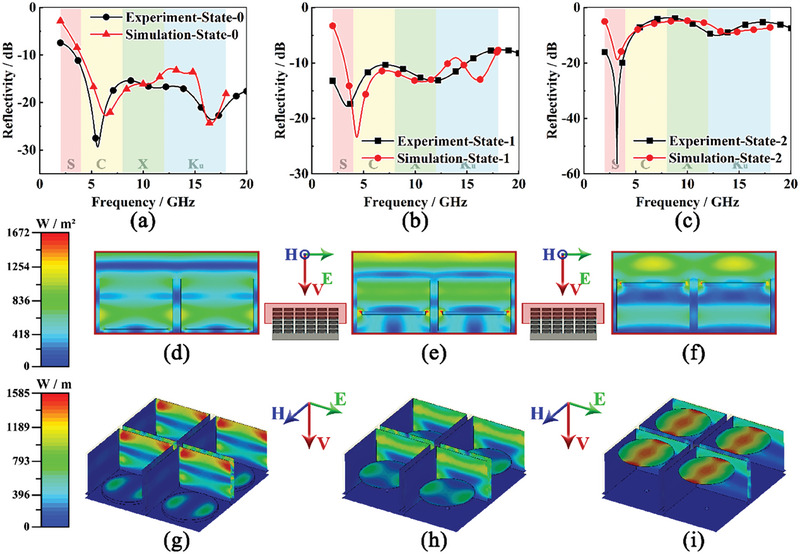
Simulation results considering the practical states: a–c) Comparison of reflectivity curves in State‐0, State‐1 and State‐2, d–f) Nephogram of the electric field in State‐0, State‐1 and State‐2, and g–i) Nephogram of surface power loss density in State‐0, State‐1, and State‐2.

To further investigate the mechanism of electromagnetic energy dissipation, corresponding simulation models are established in CST Studio Suite according to the three states of Kresling origami (Note S5, Supporting Information). The origami heights in these states are set at 0, 5, and 13 mm. The experimental and simulated reflectivity curves are first compared to testify to the reliability of the proposed models (**Figure** **5**a–c). All three groups of comparisons exhibit desirable consistencies. Some minor deviations can be found in the low‐frequency range, resulting from the dimension error of the Kresling origami height. Especially for State‐1, the actual origami height is due to mechanical interference, which is related to the fabrication process and hard to keep constant for different origami units. In simulations, the origami height is set as 5 mm, which is an average value according to the measurement. To identify the operating principle of the integrated absorber, the electric field distribution and power loss density in these three states at 16.5 GHz are calculated by numerical computations. Figure 5d–f exhibits the root mean square (RMS) of the electric field on the cross‐section of the grid unit center, representing the distribution of electromagnetic energy. In each state, the concentration of the electric field can be observed above each circular resistance piece, which illustrates that these resistance pieces succeed in interrupting the transmission of microwaves. In addition, as the circle resistance pieces rise from the bottom to the top, the centers of these concentrated regions move from the side to the center of a grid unit, which implies a variation of boundary conditions for interrupted microwaves. When the resistance pieces are located at the bottom, the grid resistance acts as an electrical conductor, resulting in a resonance on the unit side. When the pieces rise to the top of grid units, the microwaves distribute in an open space. The electric field varies according to the typical FSS with a circular pattern. Besides the electric field, energy dissipation is another important approach to analyzing the operating principle of an absorber. The surface power loss density of resistance is mainly exhibited during state switching (Figure 5d–f). In state‐0, the power loss is predominantly located at the top side of the grid resistance. The phenomenon illustrates that the resistance‐type grid structure is the main active component in microwave absorption. In State‐1, the power loss on the grid resistance is much less than in State‐0. Meanwhile, the proportion of power loss on the circular resistance pieces obviously increases. In State‐2, the circular resistance pieces eventually become the predominant power‐loss components. The power loss map testifies that the essence of microwave‐absorbing variation is the switching between two microwave‐absorbing components, which conforms to the initial design concept. In addition, it is interesting to point out that the energy dissipation mode on the circular resistance pieces remarkably varies in the rise process. At the bottom, the main energy dissipation region is scattered at two sides of the pieces in the electric field direction. However, at the top, it converts to a center band in the magnetic field direction. The variation is related to the electric field distribution (Figure 5d–f). A strong electric field directly leads to a large surface power loss density in the corresponding region. The electric field distribution and power loss density at the absorption peaks in the low‐frequency range are also calculated, which exhibits a similar variation trend with the high‐frequency (Figure S11, Supporting Information).

### 2.5 Digital Adjusting Strategy

**Figure 6:**
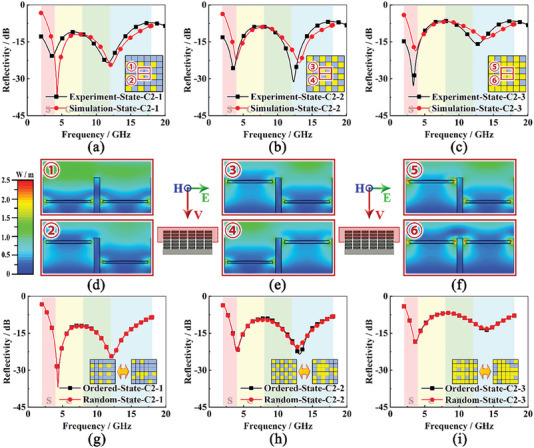
The adjusting effect of microwave absorbing performance with a programmable controlling strategy: a–c) Comparison of experimental and simulation results in three derivative states including State‐C2‐1, State‐C2‐2, and State‐C2‐3, d–f) Electric field distribution of superior units in three derivative states, and g–i) Comparison of simulation models in three derivative states with ordered and random sequences.

In the proposed design, the actuator of Kresling origami is a bistable component, implying that only two stable states are available for the integrated absorber with the restriction of all actuators in the same state. In order to expand the design flexibility, a digital adjusting strategy is applied to separately switch the state of each Kresling origami and form multiple available structural states.^[^ ^29^ ^]^ For the convenience of discussion, every 2 × 2 adjacent grid units are combined as a periodic superior unit in the following discussion. The state of each basic unit in one superior unit is independently switched between State‐1 and State‐2, while the states of all the superior units are the same. For a superior unit with 2 × 2 adjacent grid units, three derivative states are adopted with different duty ratios, denoted as State‐C2‐1, State‐C2‐2, and State‐C2‐3. The checkerboards are utilized to exhibit these states (**Figure** **6**), where the blue check represents the basic unit in State‐1 and the yellow check represents the basic unit in State‐2. The microwave absorbing performance of specimens in different derivative states is measured and exhibited (Figure 6a–c). The states of Kresling origami are adjusted manually for simple verification. It can be observed that the measured reflectivity curves possess a similar trend in the whole frequency range with two distinct absorbing peaks at nearly the same low and high frequencies (3.6 and 12.0 GHz). With increasing duty ratio, the absorbing peak in the low‐frequency range is greatly enhanced and the absorbing effect in the high‐frequency range is inversely suppressed. Consequently, the microwave absorbing performance shows a variation trend from high‐frequency broadband absorption to low‐frequency narrowband absorption, which is exactly consistent with the transition process summarized from the integrated absorber. Meanwhile, a series of entire switchable microwave absorber models with a digital adjusting strategy is established in CST Studio Suite. The radar cross section (RCS) curves of these models and the PEC background are obtained separately. Then, the reflectivity is the ratio of RCS data between each model and the PEC background. The validity of this solving method is testified by comparing with the experiment results in State‐C2‐1, State‐C2‐2, and State‐C2‐3 (Figure 6a–c). The reflectivity curves of the absorbers in State‐0, State‐1, and State‐2 are also calculated and show good consistency with experiments (Figure S12, Supporting Information).

To further investigate the microwave absorbing mechanism, RMS distributions of the electric field at the first absorbing peak are plotted (Figure 6d,e). It can be observed that the resonance forms between the impedance‐type grid structure and resistance pieces, which clearly distinguishes it from traditional FSS. In traditional FSS with circular resistive patterns, the resonance only occurs at the interval of adjacent patterns and the relative position of these adjacent resistance pieces is a critical factor to influence the electric field. When the resistance pieces are staggered in the vertical direction, the resonance disappears. However, in the proposed integrated absorber, the impedance‐type grid structure is utilized as an electrical conductor blocking interactions between adjacent patterns. As a substitute, the resonance forms between the circular resistance and grid resistance. Therefore, the electric field of each grid unit is independent of adjacent units. Only changing the arrangement but keeping the duty ratio constant renders little influence on the microwave absorbing performance.

To verify this conclusion, a series of simulation models with the same duty ratio to State‐C2‐1, State‐C2‐2, and State‐C2‐3 but random digital sequences are established. The calculated reflectivity curves are exhibited (Figure 6g–i) and the repeatability is further verified through simulations (Figure S13, Supporting Information). The comparison results show that the curves are nearly coincident in each control group. Some negligible distinctions can be found at the second absorbing peak, which mainly results from the edge effect in numerical calculations. Therefore, the duty ratio is the critical factor to adjust the microwave absorbing performance, while the arrangement renders little influence. The microwave absorber possesses excellent robustness in microwave adjusting. If parts of actuators get out of order, the other parts will be still adjusted by the pneumatic control system and achieve a similar microwave absorbing performance to the preconcerted target. In addition, the frequency of the first absorbing peak can be continuously adjusted from 5.6 to 3.4 GHz with the variation of the duty ratio. That means, by using the digital adjusting strategy, the structure can achieve a deep microwave absorbing performance at a specified frequency, which may be used for the particular absorbing demand or passive signal transmission. Furthermore, the pneumatic bistable origami and the digital adjusting strategy also provide a platform for further design, equipping with different modulators. For example, it can be designed as a tunable electromagnetic polarizer, by using strip patterns instead of circle resistance pieces.

## 3 Conclusion

Inspired by the synergistic color‐changing strategy of chameleons, we have proposed a switchable microwave absorber with ultra‐broadband stealth, taking advantage of the impedance‐type grid structure and circular resistance pieces. Moreover, Kresling origami airbags are introduced as actuators and endow the proposed device with bistable features. The geometric constraints and bistable performance are investigated through theoretical analysis and verified by FEA simulations and pneumatic experiments. The experimental results reveal that the origami structure possesses two stable states (State‐1 and State‐2) at the origami height of 10 and 28 mm, respectively. By applying an extra internal negative pressure, the origami height can eventually decrease to 4 mm (State‐0). The proactively switching points of these two states are located at the origami height of 22.5 and 20.5 mm, corresponding to the internal relative pressure of −7.6 and 14 kPa, respectively. A series of integrated microwave absorbers are fabricated according to the results of a particle swarm optimization algorithm. The electromagnetic experimental results demonstrate that the proposed device possesses complementary absorbing bands, as 4.7–18 GHz in State‐0 and 2–3.4 GHz in State‐2. The intrinsic mechanism lies in the switching of active components between impedance‐type grid and circular resistance pieces. Utilizing this strategy, we succeed in eliminating the defect of the impendence‐type grid structure in the low‐frequency range to extend the absorbing band. Furthermore, the microwave absorbing performance with a digital adjusting strategy is investigated, where basic units are controlled separately. Little coupling effect is found between adjacent units and the entire device shows extraordinary robustness. The absorbing performance is largely related to the duty ratio between two stable states, rather than the sequence.

## 4 Experimental Section

### Manufacturing process

The main part of the grid structure was fabricated through selective laser sintering (SLS) with Nylon particles (PA2200, Stratasys, 100 µm). The printing precision was ≈0.1 mm. The resistance pieces were fabricated through a screen printing process, including grid resistance and circular resistance pieces. The conductive ink was solidified on polyimide (Pi) film (25 µm, thickness), cut into the desired size with a digital cutting machine, and stuck on the device with instant adhesive (Loctite 415). The Kresling origami was fabricated manually. The polygon and triangle panels were cut from PVC plastic plates (0.5 mm, thickness) and reconnected into the Kresling origami configuration by PET film (50 µm, thickness) and instant adhesive (Loctite 415).

### Pneumatic Experiment

The Kresling origami air bag was inflated and exhausted by a syringe pump (Lead Fluid TYD01‐02). The internal relative pressure was measured by an electronic pressure gauge (Tecman TM510). The deformation process was recorded by a video recorder together with the measurement result of the pressure gauge. After experiments, the experimental data were obtained according to the video.

### Electromagnetic Experiment

The electromagnetic performance of Nylon PA2200 was measured using the waveguide method with a vector network analyzer (AV3672D, China Electronics Technology Group Corporation). Four rectangular waveguides were used in the measurement. The experimental data were analyzed and summarized into a lossless result. The microwave reflectivity was measured on a comprehensive testing platform (AV9809S, China Electronics Technology Group Corporation) with a pair of horn antennas on an arch frame. A steel flat panel was utilized as the background. Some small holes (3 mm) were drilled on the panel for the gas circuit. The frequency range was 1–18 GHz, and the number of frequency points was set as 2001. The switching between State‐1 and State‐2 was conducted manually. A vacuum pump was used to apply extra internal negative pressure to achieve State‐0. For each state, the reflectivity data were measured at least thrice.

## Conflict of Interest

The authors declare no conflict of interest.

## Supporting information

Supporting Information

Supplemental Movie 1

Supplemental Movie 2

Supplemental Movie 3

Supplemental Movie 4

Supplemental Movie 5

## Data Availability

The data that support the findings of this study are available from the corresponding author upon reasonable request.
