# Supplementary material for: Bioinspired Double‐Broadband Switchable Microwave Absorbing Grid Structures with Inflatable Kresling Origami Actuators
Source: Adv Sci (Weinh). 2023 Nov 30;11(4):2306119. doi: 10.1002/advs.202306119 (PMC10811514; doi:10.1002/advs.202306119)
Supplement: Supplementary file 1 — Supporting Information [file ADVS-11-2306119-s006.pdf]

ADVANCED SCIENCE
Open Access

Supporting Information

for *Adv. Sci.*, DOI 10.1002/advs.202306119

Bioinspired Double-Broadband Switchable Microwave Absorbing Grid Structures with Inflatable Kresling Origami Actuators

Zhong Zhang, Hongshuai Lei, Shengyu Duan*, Zeang Zhao, Mingji Chen, Changxian Wang and Daining Fang*

Supporting Information

Bioinspired double-broadband switchable microwave absorbing grid structures with inflatable Kresling origami actuators

Zhong Zhang, Hongshuai Lei, Shengyu Duan*, Zeang Zhao, Mingji Chen, Changxian Wang, and Daining Fang*

S1 Geometric Relationship in Kresling Origami

To derive the specific geometric relationship of structural parameters in the Kresling origami, a simplified constraint diagram is shown in Figure S1. In the folded state, as the side panels are congruent triangles, three adjacent triangular side panels are utilized to represent the constraint (Figure S1a), which are distinguished with different colors, i.e., $\Delta\mathrm{ABC}$ (red), $\Delta\mathrm{CFA}$ (yellow) and $\Delta\mathrm{FCE}$ (green). Therefore, the constraint can convert to an equation of $\angle\mathrm{ECB}$ as:

$$\angle\mathrm{ECB} = \angle\mathrm{ACB} - \angle\mathrm{ACF} + \angle\mathrm{ECF} \tag{S1}$$

where $\angle\mathrm{ECB}$ is the interior angle of the polygon bottom panel. According to the property of congruent triangles, $\angle\mathrm{ACF}$ equals to $\angle\mathrm{CAB}$ and $\angle\mathrm{ECF}$ equals to $\angle\mathrm{ABC}$. Therefore, Equation (S1) can be solved as:

$$\angle CAB = \frac{\pi}{n} \tag{S2}$$

where n refers to the quantity of polygon sides. According to the Cosine law, the relationship between triangle side length and polygon side quantity can be given as follows:

$$\frac{l_b^2 + l_c^2 - l_a^2}{2 l_b l_c} = cos\frac{\pi}{n} \tag{S3}$$

where l_a, l_b and l_c refer to the side length values of the triangle panel.

In the expanded state, different from the folded state, one side panel is enough to specify the configuration (Figure S1b). $\Delta\mathrm{A'BC}$ (red) represents the side panel in the expanded state, $\Delta\mathrm{DBC}$ (pink) is the projection of $\Delta\mathrm{A'BC}$ on the bottom plane, and $\Delta\mathrm{ABC}$ represents the side panel in the folded state. It should be noted that the central

axes of the top and bottom polygon are completely coincident. Therefore, points A and D are both on the circumcircle of the bottom polygon. Simultaneously, ΔDBC and ΔABC share the same side $\overline{BC}$, so that ∠BDC and ∠BAC correspond to the same arc length on the circumcircle and possess the same angle. According to the Cosine law, another equation can be established in ΔDBC and ΔABC, as follows:

$$\frac{\overline{AB}^2+\overline{AC}^2-\overline{BC}^2}{2\overline{AB}\,\overline{AC}}=\frac{\overline{DB}^2+\overline{DC}^2-\overline{BC}^2}{2\overline{DB}\,\overline{DC}} \tag{S4}$$

Considering the side length values $\overline{DB}$ and $\overline{DC}$ can be expressed through the Pythagorean theorem in ΔA′DB and ΔA′DC, Equation (S4) can be rewritten as follows:

$$\frac{l_b^2+l_c^2-l_a^2}{2l_bl_c}=\frac{l_b^2+l_c^2-l_a^2-2H_2^2}{2\sqrt{(l_b^2-H_2^2)(l_c^2-H_2^2)}} \tag{S5}$$

Combining the Equations (S3) and (S5), the relationship between the side length of the triangle panel and origami height is established. As the demand for origami height is given, the side length values can be obtained by solving simultaneous equations. Inversely, as the side length is given, the simultaneous equations can be translated as follows:

$$\frac{l_b^2+l_c^2-l_a^2-2h^2}{2\sqrt{(l_b^2-h^2)(l_c^2-h^2)}}=cos\frac{\pi}{n} \tag{S6}$$

where h refers to the undetermined origami height. Essentially, the equation is a quadratic equation with respect to h^2. The quantity of nonnegative solution of h equals to the number of stable states for the origami structure.

In addition, according to the derivation process of geometric constraint in expanded state, ∠AOD is the twist angle (θ) between stable states (Figure S1). Points A and D are both on the circumcircle of the bottom polygon. ∠AOD is the central angle of arc $\widehat{AD}$ and ∠ABD is the corresponding angle at the circumference. The angle value of ∠AOD is twice as large as ∠ABD. ∠ABD can be calculated by the Cosine law in ΔABC and ΔDBC. Therefore, the twist angle (θ) can be expressed as follows:

$$\theta=2\left(arccos\frac{l_a^2+l_c^2-l_b^2}{2l_al_c}-arccos\frac{l_a^2+l_c^2-l_b^2}{2l_a\sqrt{l_c^2-H_2^2}}\right) \tag{S7}$$

S2 Simulation Model for Single Kresling Origami

To verify the bistable feature of Kresling origami, a qualitative compressive model is established in ABAQUS (Figure 2). The polygonal terminal panels and triangle side panels are both modeled with a 3D deformable shell and assembled with “tie” constraint. To simulate a flexible connection effect between panels, boundary strips of 0.5 mm are separated from these panels. The majority of panels (blue part in Figure 2) are endowed with purely elastic PA material and a thickness of 0.5 mm, while the boundary strips (brown part in Figure 2) are endowed with purely elastic PET material and a thickness of 0.05 mm. Therefore, the flexural modulus of the connection is rather small. The origami structure can be folded along the boundary line flexibly, while maintaining a certain stiffness against the deformation of side panels. In terms of boundary conditions, the bottom polygonal panel is completely fixed and the top polygonal panel is applied with a smooth axial displacement load (total 26 mm) through a respective reference point. The whole model is meshed with a triangular shell unit (S3R). The job is calculated with a dynamic explicit solver. It should be noted that the self-contact interaction is not involved in the simulation to obtain the mechanical response under ideal conditions. Therefore, the origami model is completely compacted at the end of the analysis step. Simultaneously, some mechanical interference phenomena can be observed during the compression process.

S3 Durability Tests for Single Kresling Origami

In this investigation, the Kresling origami specimens are fabricated manually, which is mainly utilized in the electromagnetic performance verification. As the actuator of the whole device, the durability of these origami airbags is a significant property for practical applications. Therefore, a series of pneumatic tests are conducted to test the durability of these fabricated airbags. In the pneumatic tests, a newly prepared Kresling origami airbag is utilized as the specimen. The bottom terminal panel of the specimen is fixed on the background and the internal pressure is adjusted by the compressing or pulling strokes of a syringe. In the compressing stroke, the internal pressure is about 21 kPa, while in the pulling stroke, the internal pressure is about -10 kPa. After each stroke, the internal pressure is reset to 0 kPa and the origami height is recorded in the

corresponding stable state (Figure S2a and Movie S4). A total of 100 cycles is conducted on the specimen. For each 50 cycles, the origami height and internal pressure at the buckling points are measured and compared (Figure S2b and c). It can be observed that the origami height exhibits a slight fluctuation in the preliminary stage and then becomes steady. In the expanded state, the origami height exhibits a decreasing trend with a final height of about 27.5 mm. In the folded state, the origami height exhibits an increasing trend with a final height of about 9.7 mm. This performance is due to the fatigue of the creases which become more flexible after bending more times. A slight difference can be distinguished between this specimen and the specimen in Section 2.2, which mainly results from the manufacturing deviation. In addition, the origami height and internal pressure at the buckling points also exhibit a slight variation. In extraction process, the buckling origami height and internal pressure gradually increase, while in the inflation process, they gradually decrease. This phenomenon is also related to the flexibility of creases. With the flexibility increases, the barrier energy decreases, less external energy is required to switch the states of the specimen, and less structural deformation is observed at the buckling points.

S4 Particle Swarm Optimization Algorithm

To obtain a series of superior structural parameters, the particle swarm optimization algorithm is utilized and adaptively modified. The main program is compiled by MATLAB and associated with CST Studio Suite to capture the reflectivity data at each frequency (ranging from 2-18 GHz). The optimization parameters include the unit period (a), wall thickness (t), lattice sheet resistance (R_1), circular sheet resistance (R_2), circular sheet radius (r_1) and origami height (H_2). During the parameter iteration process, the data ranges of all parameters are normalized as 0-1. The quantity of particles is set as 60 and the maximum iteration is set as 30. The maximum moving speed of a single particle in one dimension is limited to 0.05. Some minor modifications have been conducted to the iteration strategy. First, the moving direction of particles in each iteration step is modified. For a typical particle swarm optimization algorithm, all of the particles move toward the generational optimum point. In this optimization, five

generational optimum points are recorded and all of the other sample particles move toward the closest generational optimum point (Figure S3a). The improvement refers to the strategy of a multi-island genetic algorithm, increasing the opportunity to obtain the global optimum point. Then, the iteration step in each iteration step is modified. For a typical particle optimization algorithm, the step length is in direct proportion to the spatial distance between the sample point and generational optimum point, resulting in a remarkable decline in convergence rate when sample particles get close to the global optimum point. In this optimization, a correction factor is introduced to slightly counteract the influence of spatial distance, so as to accelerate the iteration speed (Figure S3b).

To effectively evaluate the performance of proposed switchable microwave absorbing devices, a set of customized fitness functions is established (Figure S4). The reflectivity value at each frequency point for different configuration states is obtained from simulation and the data of each configuration state are divided into two different sets according to the frequency range (including low frequency and high frequency). The individual score is first calculated at each frequency point according to a piecewise evaluation function for the corresponding section. Particularly, the sectional evaluation function includes two reference values, denoted as the expected value (optimization objective -5 dB or -15 dB) and the base value (-10 dB). If the reflectivity is better than the expected value, it gets the full score at this frequency. Otherwise, the score gradually decreases till to zero. When reflectivity exceeds the base value, a negative score is attained. Finally, a sectional score is obtained by accumulating the individual score of each frequency point in the corresponding section, and the whole score of a configuration is the product of all sectional scores. It should be noted that the breaking point between low and high frequencies is an undetermined value in a specific range (4-6 GHz). The optimization program will find a proper breaking point to maximize the whole score of a configuration.

S5 Unit Cell Simulation Model

To predict the microwave absorbing performance of the proposed switchable microwave device in different states, a series of simulation models is established in CST Studio Suite (Figure S5). The simulation model is a single unit of the integrated absorber, including a unit of the lattice structure, a circular resistance piece and a corresponding Kresling origami structure. The main part of the model is constructed with a nondispersive medium, Nylon PA2200, and the resistance is simulated with an ohmic sheet. The electromagnetic properties of Nylon have been measured with the waveguide method. The relative permittivity is 2.64+0.02i and the relative permeability is 1.02+0.01i. The investigated frequency is set as 2-18 GHz. The boundary conditions in x- and y-directions are set as “unit cell” and in z-directions are “open (add space)”. The project is calculated with the Frequency Domain Solver. The reflectivity curves are directly obtained from the S-parameters. The simulation models are utilized as a prediction module in the optimization process. To accelerate the calculation process, the origami structure is neglected in simulation, as the thickness of origami walls (0.4 mm) is far less than the minimum wavelength (16.7 mm), and renders little influence on the reflectivity. The rationality of this simplification is verified in preliminary simulations (Figure S6).

S6 Additional Optimization Design

To make amends for neglecting the stably folded origami height, an additional optimization design is conducted with the same optimization program. Some minor parameters are changed to meet the optimization demand. The height of lattice structure (H_1) is raised to 20 mm and the origami height (H_2) in the folded state is set as 5 mm. The optimization results are concluded in Table S3 and Figure S12.

Supporting figures

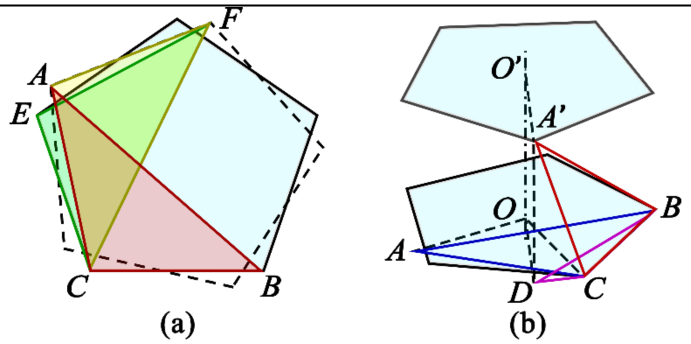

Figure S1. Schematic diagram of simplified geometric constraint in different states: a) folded state and b) expanded state.

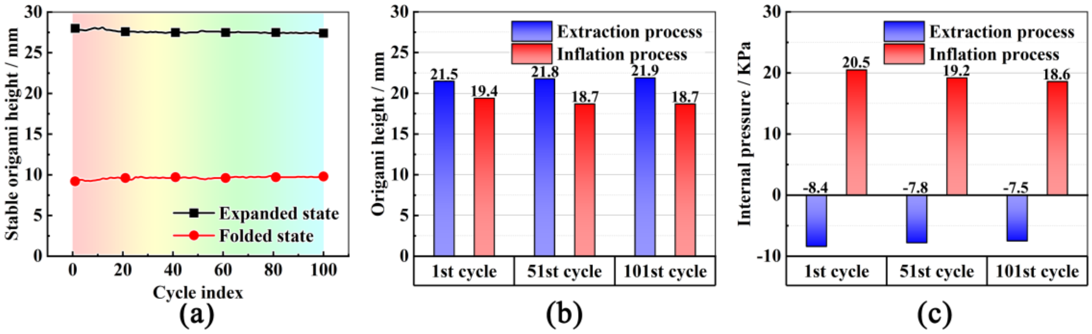

Figure S2. Durability performance of single Kresling origami airbags: a) stable origami height, b) buckling origami height, c) buckling internal pressure.

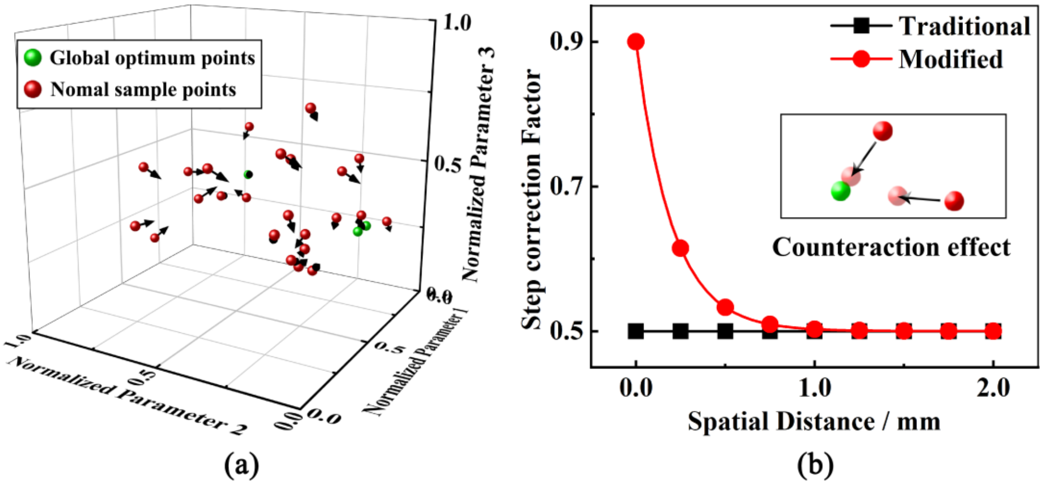

Figure S3. Modified particle swarm optimization algorithm: a) schematic diagram of the iteration with multiple global optimum points and b) iteration step length correction according to the spatial distance.

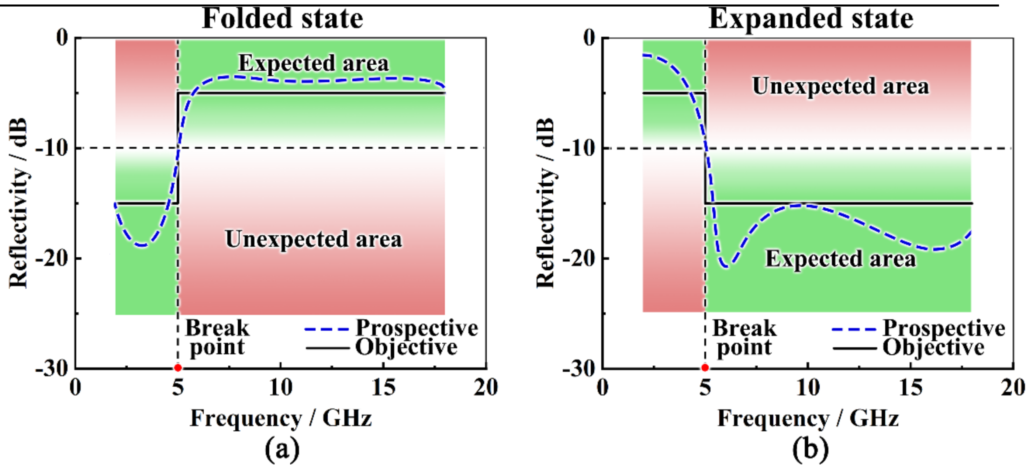

Figure S4. Customized evaluation functions for the microwave absorbing performance in different states: a) folded and b) expanded states.

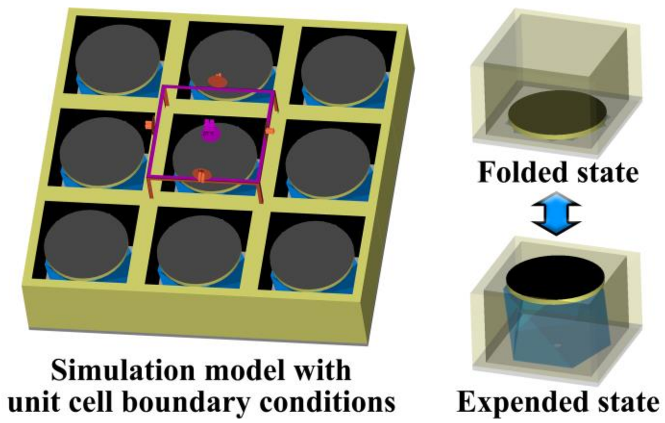

Figure S5. Unit cell simulation model of switchable microwave absorber in CST Studio Suite.

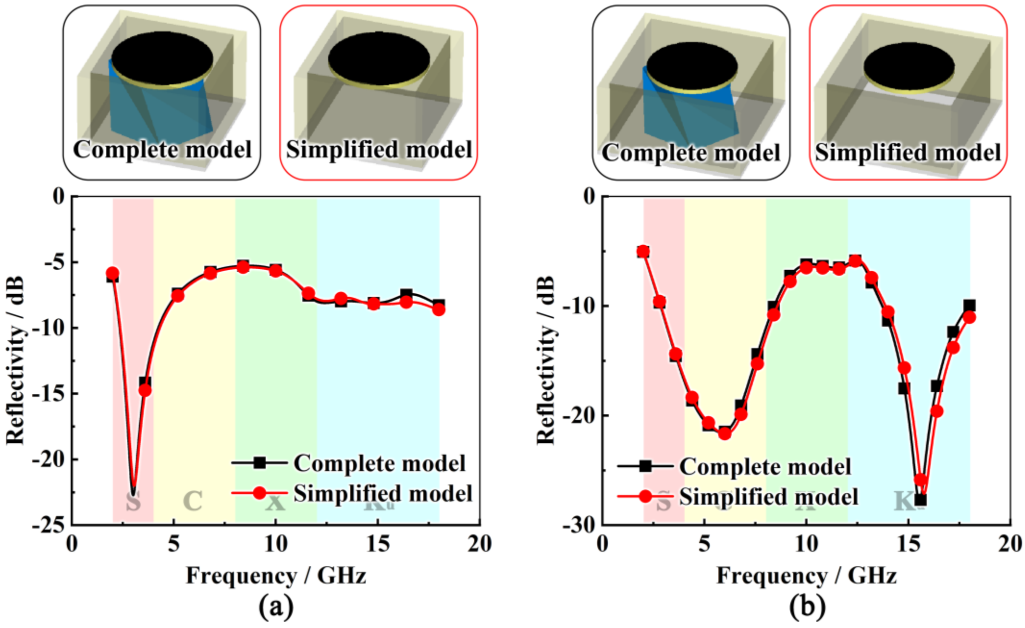

Figure S6. Comparison of reflectivity curves between complete and simplified model with two sets of structural parameters.

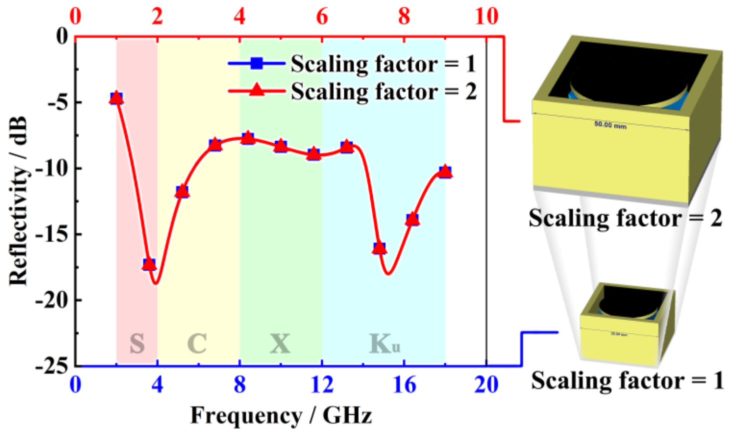

Figure S7. Comparison of microwave absorbers with different scaling factor.

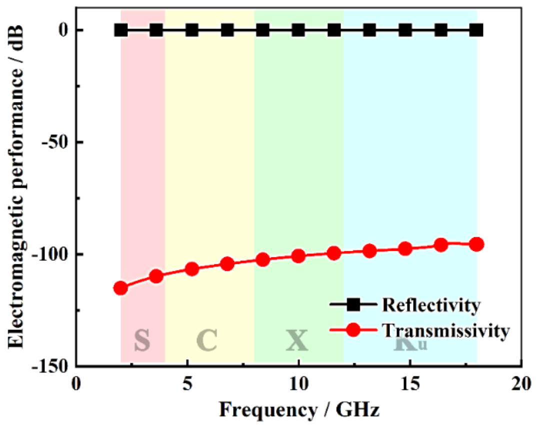

Figure S8. Electromagnetic performance of steel flat backboard with small holes.

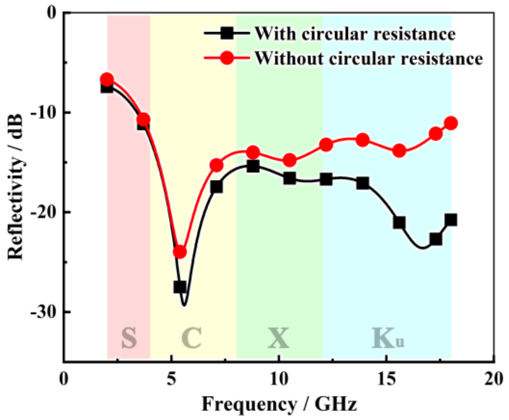

Figure S9. Comparison of microwave absorbing performance between impedance-type lattice structure in State-0 with and without circular resistance pieces.

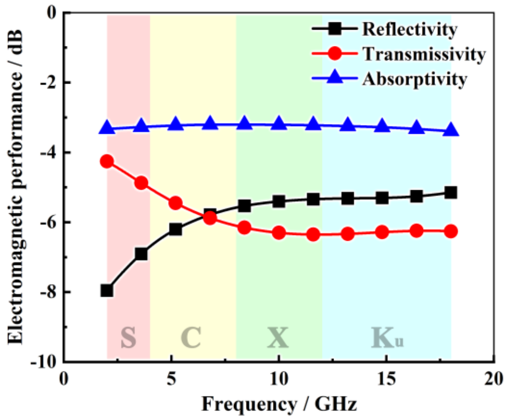

Figure S10. Electromagnetic performance of circular resistance pieces.

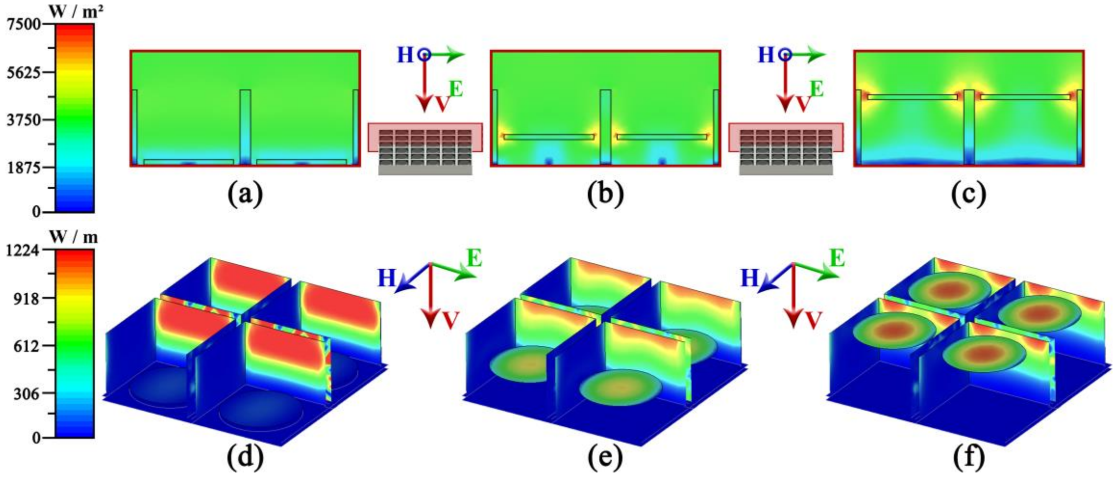

Figure S11. Simulation results at low-frequency absorption peaks: a-c) Nephogram of the electric field in State-0, State-1 and State-2 and d-f) Nephogram of surface power loss density in State-0, State-1 and State-2.

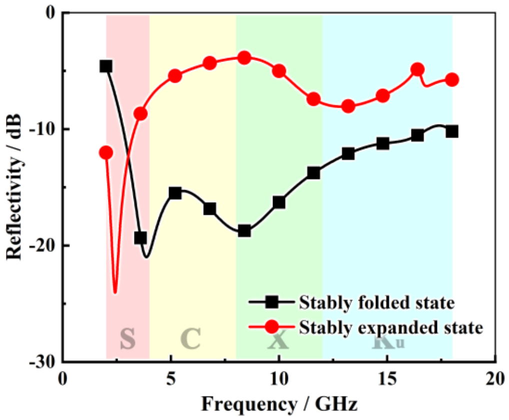

Figure S12. Additional optimization result considering the stably folded origami height.

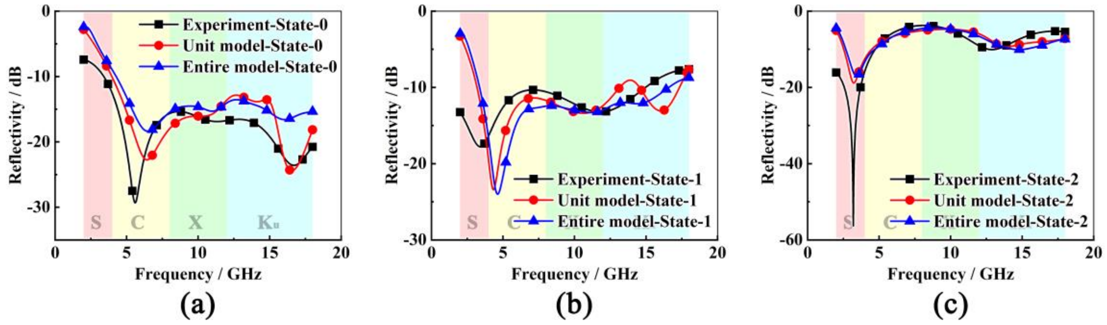

Figure S13. Comparison between unit and entire simulation models in State-0, State-1 and State-2.

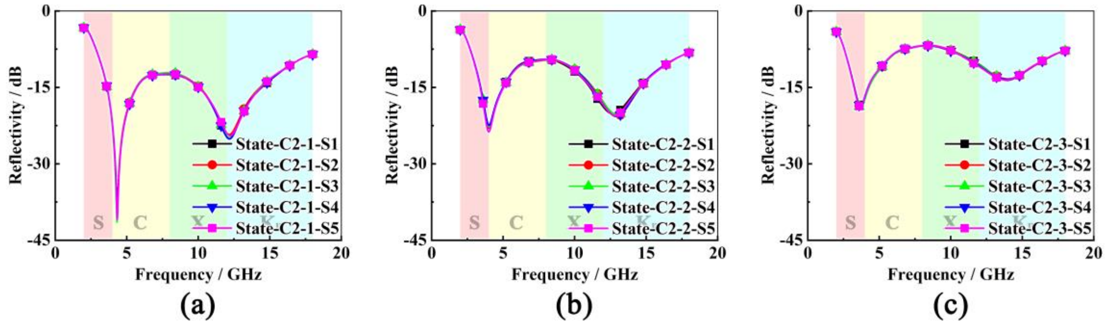

Figure S14. Repeatability tests of simulation models with random sequence and the same duty ratio: a) State-C2-1, b) State-C2-2 and c) State-C2-3.

Table S1. Structural parameters of Kresling origami for FEM simulation analysis.

Polygon side quantity (n)	Circumcircle radius (r_2) [mm]	Origami height (H_2) [mm]	Triangle side length $\overline{BC}$ (l_a) [mm]	Triangle side length $\overline{AC}$ (l_b) [mm]	Triangle side length $\overline{AB}$ (l_c) [mm]
5	20.0	26.0	23.53	27.14	39.22

Table S2. Primary designed structural parameters using particle swarm optimization.

Optimized structural parameters					
Unit period (a) [mm]	Wall thickness (t) [mm]	Lattice sheet resistance (R_1) [Ω sq^{-1}]	Circular sheet resistance (R_2) [Ω sq^{-1}]	Circular piece radius (r_1) [mm]	Origami height (H_2) [mm]
22.5	2.25	349.0	99.0	8.9	13.0

Other structural parameters					
Lattice height (H_1) [mm]	Circumcircle radius (r_2) [mm]	Polygon side quantity (n)	Triangle side length $\overline{BC}$ (l_a) [mm]	Triangle side length $\overline{AC}$ (l_b) [mm]	Triangle side length $\overline{AB}$ (l_c) [mm]
15.0	10.0	5	11.76	13.57	19.61

Table S3. New optimized structural parameters considering the folded origami height.

Optimized structural parameters					
Unit period (a) [mm]	Wall thickness (t) [mm]	Lattice sheet resistance (R_1) [Ω sq^{-1}]	Circular sheet resistance (R_2) [Ω sq^{-1}]	Circular piece radius (r_1) [mm]	Origami height (H_2) [mm]
20.1	0.5	698.8	108.8	9.6	15.0
Other structural parameters					
Lattice height (H_1) [mm]	Circumcircle radius (r_2) [mm]	Polygon side quantity (n)	Triangle side length $\overline{BC}$ (l_a) [mm]	Triangle side length $\overline{AC}$ (l_b) [mm]	Triangle side length $\overline{AB}$ (l_c) [mm]
20.0	10.0	5	11.76	15.10	19.92

Movie S1. Deformation process in air extraction tests. In order to analyze the mechanical performance, the deformation process of a single origami airbag is recorded in an air extraction test.

Movie S2. Deformation process in air inflation tests. In order to analyze the mechanical performance, the deformation process of a single origami airbag is recorded in an air inflation test.

Movie S3. Deformation process in pressure recovery tests. In order to analyze the mechanical performance, the deformation process of a single origami airbag is recorded in a pressure recovery test.

Movie S4. Deformation process in pneumatic cycle tests. As a reference for the durability performance, the deformation process of a single origami airbag is recorded in a pneumatic cycle test.

Movie S5. Switching process in pneumatic tests. As a reference for the pneumatic performance, the switching process of a whole assembled microwave device is recorded in a pneumatic test.
